# Genetic Evidence of *SpGH9A3* in Leaf Morphology Variation of *Spathiphyllum* ‘Mojo’

**DOI:** 10.3390/genes15091132

**Published:** 2024-08-28

**Authors:** Songlin Yang, Minghua Hu, Runxin Wu, Zhiwen Hou, Huan Zhang, Wenying He, Lili Gao, Feixiong Liao

**Affiliations:** Guangdong Key Laboratory for Innovative Development and Utilization of Forest Plant Germplasm, College of Forestry and Landscape Architecture, South China Agricultural University, Guangzhou 510642, China; lsyangyn@163.com (S.Y.); 17875304754@163.com (W.H.);

**Keywords:** cellulose, cellulase, glycoside hydrolase 9, leaf

## Abstract

Leaves play a crucial role as ornamental organs in *Spathiphyllum*, exhibiting distinct differences across various *Spathiphyllum* varieties. Leaf development is intricately linked to processes of cell proliferation and expansion, with cell morphology often regulated by plant cell walls, primarily composed of cellulose. Alterations in cellulose content can impact cell morphology, subsequently influencing the overall shape of plant organs. Although cellulases have been shown to affect cellulose levels in plant cells, genetic evidence linking them to the regulation of leaf shape remains limited. This study took the leaves of *Spathiphyllum* ‘Mojo’ and its somatic variants as the research objects. We screened four cellulase gene family members from the transcriptome and then measured the leaf cellulose content, cellulase activity, and expression levels of cellulase-related genes. Correlation analysis pinpointed the gene *SpGH9A3* as closely associated with leaf shape variations in the mutant. Green fluorescent fusion protein assays revealed that the SpGH9A3 protein was localized to the cell membrane. Notably, the expression of the *SpGH9A3* gene in mutant leaves peaked during the early spread stage, resulting in smaller overall leaf size and reduced cellulose content upon overexpression in *Arabidopsis*.

## 1. Introduction

The shape and structure of leaves play a crucial role in plant growth, directly impacting photosynthetic efficiency, gas exchange rate, and overall growth conditions. Plant leaf growth and development are intricate processes influenced by numerous genetic factors [1]. Understanding how cell proliferation and expansion contribute to the formation of complex organs, and how these processes are coordinated in time and space during pattern formation, are key questions in developmental biology [2]. Key structural characteristics of plant leaves include leaf base, edge, tip, and veins. Researchers have conducted comprehensive studies on various aspects such as appearance characteristics, internal organization, and cell structure [3]. Genetic research on model plants such as *Arabidopsis thaliana* has significantly contributed to our comprehension of the intricate process of leaf development. The utilization of bioinformatics, modern molecular biology, omics analysis, and other methodologies has enhanced the understanding of molecules involved in plant leaf development and morphogenesis mechanisms.

The morphogenesis of plant organs is closely related to processes such as growth and development, cell proliferation, and cell expansion, and these processes are regulated by the cell wall. Cellulose is the main component of plant cell walls. It is synthesized in the Golgi apparatus by the synthase complex and transported to the cell membrane under the action of related proteins to complete the basic synthesis of cellulose. The cellulose metabolic process is precisely regulated by a series of synthetases, hydrolases, and modification enzymes [4]. The *glycoside hydrolase 9* gene family encodes cellulases that target β-1,4-glucan polymerization by breaking endo-β-1,4 glycosidic bonds between glucose groups within the polysaccharide chain (cellulose). Cellulase activity and expression levels may regulate cellulose biosynthesis by affecting cell expansion and division, plant mechanical strength, resistance to pathogens, and other aspects, thereby changing the composition and function of plant cells [5]. *KORRIGAN* (*KOR*), belonging to the *GH9A* subclass, is believed to impact cellulose biosynthesis and/or deposition in plant cell walls [6]. *KOR1* plays a crucial role in primary and secondary cell wall formation in *Arabidopsis*; suppressing *KOR* gene expression results in *Arabidopsis* mutants displaying dwarf phenotypes and cell wall abnormalities [7]. Upregulation of the *PttCel9A1* gene can enhance the amorphous cellulose content in transformed *Arabidopsis* plants and notably decrease cellulose crystallinity [8]. Cellulases also impact cellulose content in the cell walls of plants such as *Arabidopsis*, rice, poplar, and tomato [8,9,10,11,12]. Mutations in the *Arabidopsis* endoglucanase gene can result in significant leaf and organ shrinkage, abnormal cell wall structure, and alterations in overall plant morphology [13]. While these studies offer valuable insights into how cell wall-related functional genes influence phenotype by affecting cell number or size, there is still a scarcity of relevant research in ornamental plants.

*Spathiphyllum*, a perennial plant from the Araceae family, is native to Central and South America and Southeast Asia. It is recognized for its various leaf shapes, including lanceolate, oval, spoon-shaped, and egg-shaped, with prominent veins, long petioles, and glossy green to dark green leaves, making it highly ornamental. By the early 20th century, *Spathiphyllum* had become popular as a potted ornamental plant, playing a significant role in the global indoor foliage plant market. The growing demand for diverse leaf characteristics and new *Spathiphyllum* varieties has led to research efforts in traditional breeding methods such as screening for distinct traits, hybridization, and polyploid mutagenesis [14]. Some studies have also explored breeding through somatic cell hybridization [15,16,17]. While molecular biology and genomic technologies have revolutionized modern plant breeding, their application in ornamental plants such as *Spathiphyllum* is still limited. Recent research has demonstrated that the use of SSR and core primers can effectively differentiate *Spathiphyllum* germplasm resources and accurately identify hybrids, thereby expediting the breeding process and improving reliability [18]. The discovery of numerous SSR and SNP sites through the screening of *Spathiphyllum* leaf transcriptome data has provided valuable insights [19]. However, there is still a lack of understanding regarding the gene regulation mechanisms that govern leaf growth and development in *Spathiphyllum*.

As a foliage plant, the leaf traits of *Spathiphyllum* are essential for breeding and cultivation. Changes in *Spathiphyllum* leaves mainly involve size, length, and thickness. Despite qualitative descriptions and variety analysis, limited research has focused on the formation and growth of *Spathiphyllum* leaves. In a recent study by Li et al. [20], researchers explored post-budding leaf growth, developed a growth model using the Logistic growth curve equation, and identified key time nodes and growth characteristics. By segmenting growth time based on this model, the research team conducted transcriptome sequencing of *Spathiphyllum* leaves at different stages [19]. Transcriptome analysis revealed significant differences in cellulase expression during leaf growth in various *Spathiphyllum* varieties. This raises the following questions: do cellulase-related genes influence the leaf morphology of *Spathiphyllum*? Furthermore, how do these genes regulate the morphology of *Spathiphyllum* leaves? This study integrated the results of cellulose content and cellulase activity in *Spathiphyllum* leaves across three expansion stages, identifying *SpGH9A3* closely associated with leaf morphological development. Cellulase likely plays a vital role in leaf expansion by regulating processes such as cell wall formation, impacting cell division, and overall volume growth. Investigating the functions of the candidate gene provides valuable insights into the role of cellulases and related genes in the development of morphological differences in *Spathiphyllum* leaves. Ultimately, this research contributes to understanding the regulatory mechanism of *Spathiphyllum* leaf growth.

## 2. Materials and Methods

### 2.1. Plant Materials

*Spathiphyllum* ‘Mojo’-Ssm-1 is a somatic mutant variety of *Spathiphyllum* ‘Mojo’, derived from bud mutation selection. We conducted tissue propagation of the bud mutants and subsequently transplanted some of the resulting seedlings to the greenhouse for cultivation. The *Spathiphyllum* ‘Mojo’(S) and its somatic variants (*Spathiphyllum* ‘Mojo’-Ssm-1)(T) were grown in a greenhouse under the following conditions: at a natural light intensity of 1500 μmol/m^2^s for 14 h/10 h (light/dark) photoperiod and temperature of 23 ± 2 °C. *Spathiphyllum* leaf buds are formed at the base of the leaves and are wrapped by membranous sheaths. As the buds grow, the leaves are exposed from the sheaths. Subsequently, the expansion process of the *Spathiphyllum* leaf was divided into three stages: (1) the curled leaf stage, (2) the early spread stage, and (3) the late spread stage according to the study by Li et al. [20] (Table 1). In this experiment, the leaf of *Spathiphyllum* ‘Mojo’ was used as the control group (wild), and the leaf of *Spathiphyllum* ‘Mojo’-Ssm-1 was used as the treatment group (mutant). Leaf samples were used to measure leaf indicators, cellulose content, and cellulase activity, and perform transcriptome sequencing to screen for differential genes, as well as RNA extraction and cDNA reverse.

*Arabidopsis* was used for heterologous transgene verification, while *Nicotiana benthamiana* was used for subcellular localization of the target gene. *Arabidopsis* seeds were treated with 75% ethanol and 1% sodium hypochlorite for 30 s and 1 min, respectively, then washed with sterile water several times before sowing in 1/2 MS medium. The seeds were incubated in the culture chamber for 7 days and then transferred to nutrient soil and infected when the inflorescences emerged. *Nicotiana* seeds were sown in nutrient soil and injected with bacterial solution after they grew into young leaves. The infected *Nicotiana* leaves were placed into glass slides and observed under a confocal laser microscope for observation and imaging. The culture conditions of both plant materials were 25 ºC/20 ºC and 16 h/8 h (light/darkness).

The strains necessary for the construction of the expression vector include the DH5α Competent *E. coli* strain (C502; Vazyme) and GV3101 *Agrobacterium tumefaciens* strain (LM12-162; LMAI). DH5α is commonly used to transform plasmids into competent *E. coli* cells. The methodology is designed to maximize the efficiency of transformation, crucial for subsequent molecular biology experiments such as gene cloning and expression analysis [21]. *A. tumefaciens* strain GV3101 is widely utilized for plant transformation. The T-DNA binary vector system in *A. tumefaciens* is instrumental in facilitating this transformation process. T-DNA is engineered by removing oncogenes and crown gall synthase genes, which enhances the efficiency of gene transfer from *A. tumefaciens* to plants. This modification effectively disarms toxic strains, thereby preventing tumor induction [22]. The reagents and equipment used in this experiment are detailed in Supplementary Tables S2 and S3.

### 2.2. Morphological Traits of Leaves Measurement

Mature leaves from two varieties of *Spathiphyllum* were randomly selected for measurement. Five plants were sampled, and 3 to 5 leaves from each plant were measured. The leaf traits that were measured included leaf length, leaf width, petiole length, plant height, and crown width, all of which were measured using a ruler. Leaf area was calculated using Image-J (version 1.8.0) software.

### 2.3. Determination of Leaf Cellulose Content and Cellulase Activity

The determination of cellulose content and cellulase activity was completed using the cellulose (CLL) content detection kit (BC4280; Solarbio) and cellulase (CL) activity detection kit (BC2545; Solarbio) provided by Beijing Solarbio Science & Technology Co., Ltd. (Beijing, China).

### 2.4. Analysis of Differential Gene and Quantitative Real-Time PCR Assays

Two plant leaf samples corresponding to the expansion period were collected and frozen in liquid nitrogen. Full-length transcriptome sequencing was completed by Beijing Biomarker Technologies Co., Ltd. (Beijing, China). The criteria for screening differentially expressed genes were a false discovery rate (FDR) of less than 0.01 and a fold change (FC) of 2 or greater. This was followed by GO function and KEGG enrichment analyses. The TBtools (version 2024.1.11) software was used to create a heatmap of the differentially expressed genes [23]. Candidate genes identified through screening were numbered in accordance with their gene ID order in the transcriptome.

Total RNA from leaves of *Spathiphyllum* was isolated using an RNA extraction kit (IVD3020-S-48; Guangzhou Magen Biotechnology Co., Ltd., Guangzhou, China). A commercially available kit (R312; Nanjing Vazyme Biotechnology Co., Ltd., Nanjing, China) was used for reverse transcription of *Spathiphyllum* RNA. Full-length *SpGH9s* cDNA sequence was isolated from *Spathiphyllum* using specific primers based on *SpGH9s* sequence in the *Spathiphyllum* transcriptome.

Quantitative real-time PCR (qPCR) assays were performed according to Liu et al. [24]. Analyses were conducted following the minimum information for publication of qPCR experiments guidelines. *Spathiphyllum TUB* genes were used as internal controls for qPCR analysis. The data reported in the results represent the relative expression values calculated by the 2^−ΔΔCt^ approximation method. All experiments were performed with three biological replicates and three technical replicates.

### 2.5. Correlation Analysis

The cellulase gene expression results of differential genes obtained from transcriptome screening were correlated with the cellulose content and cellulase activity measurement results (*p* < 0.05). The gene most closely associated with morphological differences in the mutant leaf samples was selected as the candidate gene for subsequent bioinformatics analysis and functional validation.

### 2.6. Sequence Analysis

Alignments were performed, and a phylogenetic tree was generated using the DNAMAN (version 7.0) and MEGA (version 8.0) software. The physicochemical properties of the target protein were analyzed using ProtParam. Signal peptide analysis of the gene was conducted using the online software SignalP (version 5.0). The secondary and tertiary structures of the protein were predicted using Sopm and Swiss-Model, respectively.

### 2.7. Subcellular Localization

Using specific primers, a *SpGH9A3* (GenBank: WYV98059.1) full-length cDNA sequence was inserted into the GFP N-terminus of the pC18 vector. The construct was sequenced to ensure that coding sequences were fused in the frame and no mutations occurred. The construct was transformed into *A. Tumefaciens* (strain GV3101), which was then infiltrated into *Nicotiana* leaf epidermal cells. After 48 h of incubation under a 16 h light/8 h dark cycle at 25 °C, the GFP fluorescence signal was observed under a confocal microscope.

### 2.8. Phenotypic Analysis of Transgenic Plants

35S::CAMBIA1300-*SpGH9A3* was constructed by amplifying the full length of *SpGH9A3* using specific primers and cloning it into the pCAMBIA1300 vector. This construct was introduced into *A. tumefaciens* (strain GV3101) and transformed into *Arabidopsis* (Col-0) using the floral dip method. For gene overexpression experiments, 10 *Arabidopsis* plants were inoculated with the vector. The inoculated plants were grown under greenhouse conditions as aforementioned.

Leaf samples from both the wild-type and transgenic plants were collected for quantitative real-time PCR analysis. The phenotypic differences of the natural extension state of T2 generation transgenic *Arabidopsis* in terms of leaf length, leaf width, leaf area, petiole length, pod length, and plant height were photographed and compared with the wild-type.

### 2.9. Statistical Analysis

The presented data were statistically analyzed using GraphPad Prism (version 9.0.0) software. The significance of the data was investigated through a *t*-test or analysis of variance (ANOVA) (*, *p* < 0.05; **, *p* < 0.01; ***, *p* < 0.001; ****, *p* < 0.0001).

## 3. Results

### 3.1. Comparison of the Leaf Morphologies of Spathiphyllum ‘Mojo’ (Wild) and ‘Mojo’-Ssm-1 (Mutant)

**Figure 1:**
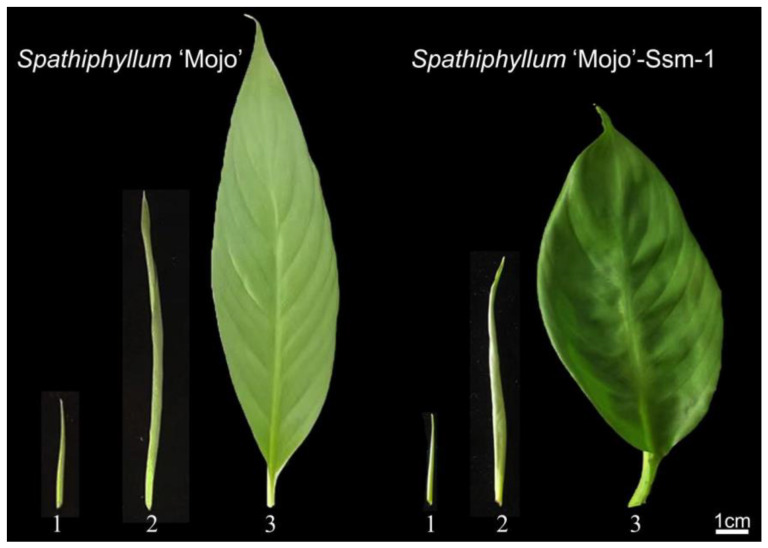
Comparison of leaf morphologies between *Spathiphyllum* ‘Mojo’ (wild) and ‘Mojo’-Ssm-1 (mutant). The expansion process of *Spathiphyllum* leaves was divided into three stages: (1) the curled leaf stage, (2) the early spread stage, and (3) the late spread stage (same as below).

*Spathiphyllum* ‘Mojo’ produces light green, lanceolate-shaped leaves. The leaf has a neat margin, an elongated tip, and a relatively narrow leaf base. ‘Mojo’-Ssm-1, on the other hand, has ovate, dark green leaves. The leaf has a tail-like tip and frequently has a curved surface (Figure 1). In terms of petiole length, plant height, leaf area, length-width ratio, and crown width, *Spathiphyllum* ‘Mojo’ considerably outperforms the mutant plants; however, its leaf width is noticeably smaller (Supplementary Table S1). The findings of the analysis show that the morphology of the leaves of *Spathiphyllum* ‘Mojo’ and ‘Mojo’-Ssm-1 differs significantly (Figure S1).

### 3.2. Comparison of Cellulose Content and Cellulase Activity

**Figure 2:**
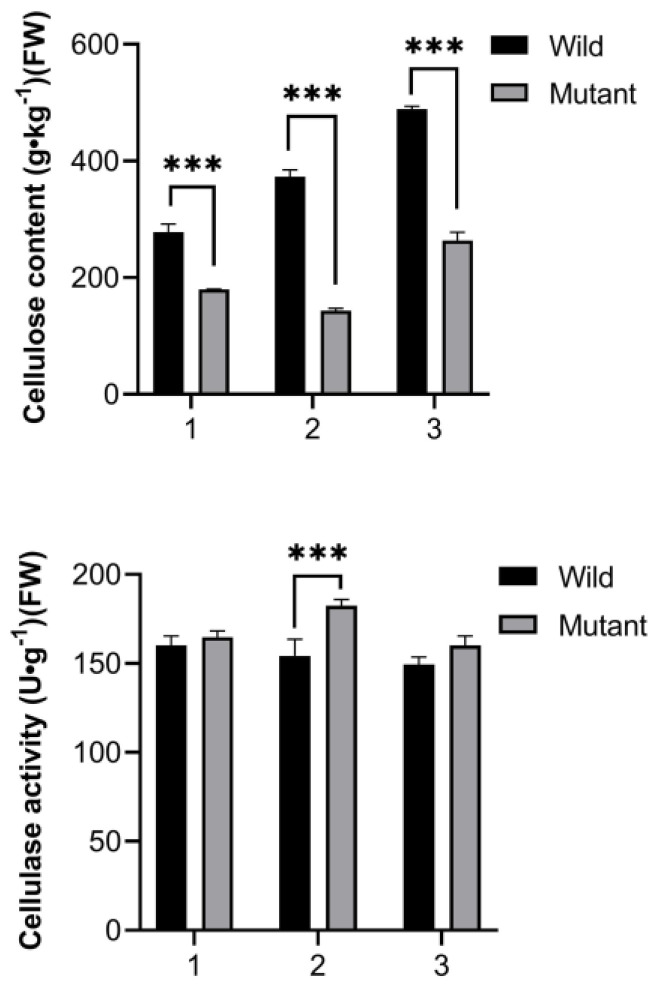
Changes in cellulose content and cellulase activity of leaves across different expansion stages. (1)–(3) represent the curled leaf stage, the early spread stage, and the late spread stage, respectively. The asterisks indicate different levels of significant differences between clusters (***, *p* < 0.001).

During the growth and development of leaves, the cellulose content in wild leaves continued to increase and was significantly higher than in mutants. The cellulose content in mutants reached its lowest value in the early spread stage, showing a trend of initially decreasing and then increasing. The cellulase activity in wild leaves gradually decreased, whereas the cellulase activity in mutants first increased and then decreased, peaking in the early spread stage. Cellulase plays a crucial role in the synthesis of cellulose essential for the expansion of different types of *Spathiphyllum* leaves, underscoring its importance in leaf growth and morphological development (Figure 2).

### 3.3. Screening of Differential Genes for Leaf Morphology

In transcriptome, 20,499 DEGs in all had KEGG annotation. Sixty-six DEGs associated with pathways such as photosynthetic, carbon metabolism, peroxidase, plant hormone signal transduction, and starch and sucrose metabolism were enriched during the curled leaf stage. Two hundred DEGs associated with pathways such as starch and sucrose metabolism, plant-pathogen interaction, endoplasmic reticulum protein processing, and spliceosome were found during the early spread stage. One hundred and sixteen DEGs that are involved in pathways such as the metabolism of starch and sucrose, the interaction between plants and pathogens, the processing of proteins in the endoplasmic reticulum, spliceosomes, and plant hormone signal transduction were found to be enriched during the late spread stage. The starch and sucrose metabolism pathway’s genes were notably enriched at all three stages, with the early spread stage showing significant enrichment, and the late spread stage showing the highest level of enrichment, far exceeding other pathways (Figure S2).

**Figure 3:**
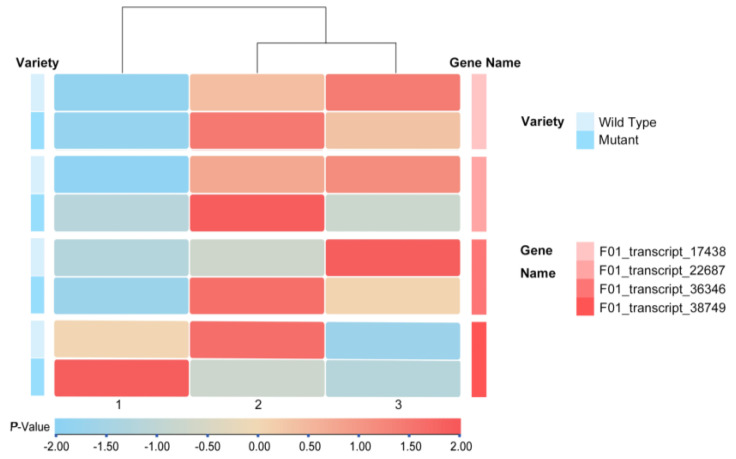
Heatmap of *GH9*s gene expression in *Spathiphyllum* ‘Mojo’ (wild) and ‘Mojo’-Ssm-1 (mutant) at three leaf expansion stages.

Four cellulase genes were analyzed in starch and sucrose metabolic pathways, revealing distinct expression patterns in different leaf types. F01_transcript_17438, F01_transcript_22687, and F01_transcript_36346 (GenBank: WYV98059.1) exhibited a gradual increase in expression levels in wild-type leaves, while in mutants, their expression initially rose and then declined, peaking during early spread stage. Conversely, F01_transcript_38749 showed significantly higher expression in mutant leaves during the curled leaf stage compared to wild-type leaves, with expression levels gradually decreasing (Figure 3).

**Figure 4:**
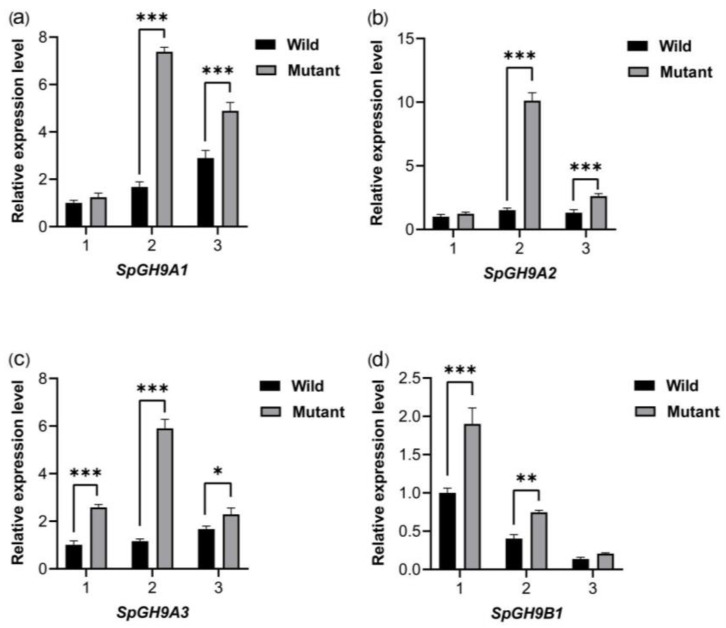
Expression patterns of *SpGH9s* across different expansion stages determined using quantitative real-time PCR. (**a**–**d**) respectively correspond to the expression levels of SpGH9A1, SpGH9A2, SpGH9A3 and SpGH9B1 in the leaves of the two varieties at different stages. The asterisks indicate different levels of significant differences between clusters (*, *p* < 0.05; **, *p* < 0.01; ***, *p* < 0.001).

Fluorescence quantitative PCR not only validated the reliability of the transcriptome data but also highlighted the expression variances of these genes in the two leaves at different expansion stages. During the early spread stage, the expression levels of F01_transcript_17438, F01_transcript_22687, and F01_transcript_36346 were significantly higher in the mutant compared to the wild-type. In the wild-type, the expression levels of F01_transcript_17438 and F01_transcript_36346 exhibited a gradual increase, while the expression level of F01_transcript_22687 remained relatively stable. On the other hand, F01_transcript_38749 showed a peak expression at the curled leaf stage, followed by a gradual decrease as the leaves expanded. These findings indicate notable differences in gene expression between the wild-type and mutant at various expansion stages, with the most pronounced disparities observed during the early spread stage. Such differences in gene expression could potentially play a crucial role in regulating leaf morphology in *Spathiphyllum* (Figure 4).

These four genes are annotated as cellulases in the transcriptome and are classified under the *GH9* gene family. To compare, we retrieved the *GH9* family amino acid sequences of *Arabidopsis* and *Oryza sativa L.* from NCBI (National Center for Biotechnology Information) and compared them with the amino acid sequences of the *Spathiphyllum GH9* family. Then, a phylogenetic tree was constructed using the NJ (Neighbor-Joining) method in MEGA 7 (version 7.0) software with 1000 bootstrap replicates. *GH9A*, *GH9B*, and *GH9C* are the three subfamilies into which the *GH9* family genes can be broadly subdivided. The *GH9A* subfamily in *Spathiphyllum* is comprised of the genes F01_transcript_17438, F01_transcript_22687, and F01_transcript_36346, whereas the *GH9B* subfamily is made up of F01_transcript_38749. F01_transcript_17438 was named *SpGH9A1*, F01_transcript_22687 was named *SpGH9A2*, F01_transcript_36346 was named *SpGH9A3*, and F01_transcript_38749 was named *SpGH9B1*, according to the gene ID order in the transcriptome of *Spathiphyllum*.

**Figure 5:**
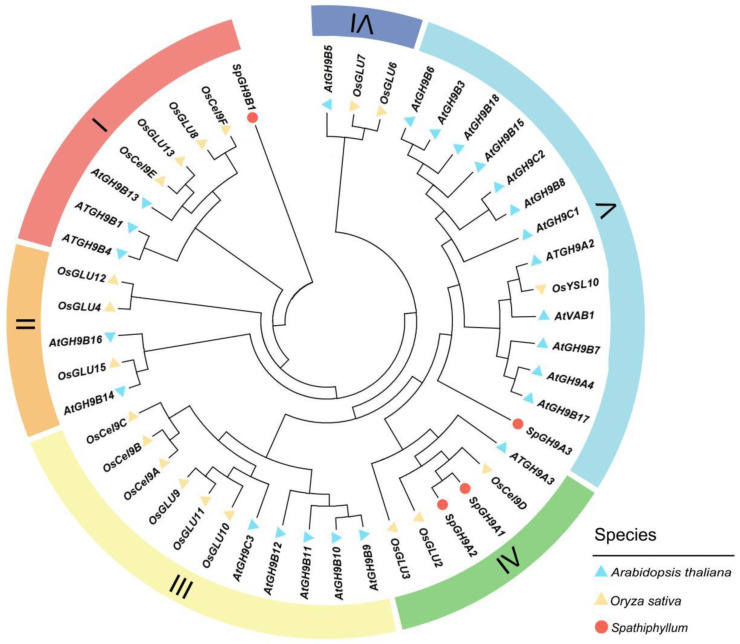
Molecular phylogenic tree of glycoside hydrolase family 9 in three plant species, including *Spathiphyllum*.

To investigate the phylogenetic relationships of the *SpGH9s* gene family members in *Spathiphyllum*, we selected the *GH9* gene families of *Arabidopsis* and *O. sativa* as references and jointly constructed a phylogenetic tree. The findings demonstrated the considerable diversity of *Arabidopsis*, *O. sativa*, and *Spathiphyllum* genes by demonstrating their intermixture and lack of independent clustering branches. Three groups comprised the four *SpGH9s* proteins. Among them, *SpGH9A1* and *SpGH9A2* belonged to group IV, *SpGH9B1* to group I, and *SpGH9A3* to group V. The support for the phylogenetic relationship between the homologous genes of *Arabidopsis* and *O. sativa* and members of the *SpGH9s* gene family in *Spathiphyllum* was relatively low, indicating that these *GH9s* members may have developed new functions during the course of evolution (Figure 5).

This study investigated the relationship between cellulose content, cellulase activity, and the expression level of *GH9s* genes, which encode cellulose endoglucanase, in the leaves of wild and mutant plants. The results revealed significant or extremely significant correlations between these parameters in both types of leaves. The correlation coefficients were 0.9690** for *SpGH9A1*, 0.8075** for *SpGH9A3*, and 0.9422** for *SpGH9B1* in wild plants, indicating a highly significant correlation with cellulose content. On the other hand, *SpGH9A2* showed a significant correlation with a coefficient of 0.4424*. Interestingly, cellulase activity and *GH9* gene expression did not show a significant correlation.

**Figure 6:**
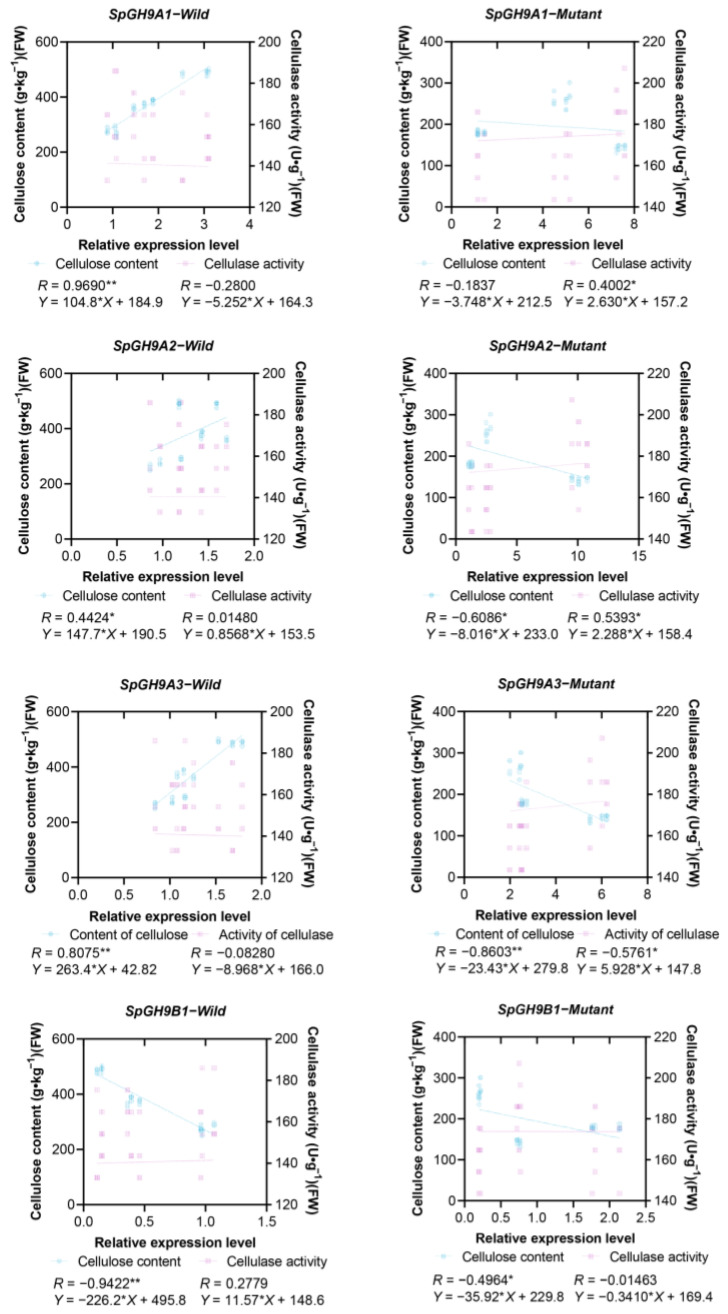
Correlation analysis of *SpGH9s* gene expression levels and cellulose content and cellulase activity of *Spathiphyllum* leaves at three leaf expansion stages. The asterisks indicate different levels of significant differences between clusters (*, *p* < 0.05; **, *p* < 0.01).

The study examined the correlation between *GH9* gene expression levels in the mutant and cellulose content. Results showed that *SpGH9A3* had a highly significant negative correlation (correlation coefficient of −0.8603**) with cellulose content, while *SpGH9A2* and *SpGH9B1* had significant negative correlations (correlation coefficients of −0.6086* and −0.4964*, respectively). However, the correlation of *SpGH9A1* (correlation coefficient of −0.1837) with cellulose content was not statistically significant. Additionally, there were varying degrees of correlation between cellulase activity and *GH9* gene expression levels. *SpGH9A1*, *SpGH9A2*, and *SpGH9A3* showed significant correlations with cellulase activity, with correlation coefficients of 0.4002*, 0.5393*, and −0.5761*, respectively. On the other hand, the correlation coefficient between *SpGH9B1* and cellulase activity was −0.01463, indicating no significant relationship. Further analysis revealed significant correlations between the cellulose content of both leaf types and the expression levels of these genes. This suggests that increased cellulase activity may lead to reduced cellulose content in the mutant, potentially impacting leaf growth and morphogenesis. Notably, all three genes from the *SpGH9As* subfamily showed significant correlations with cellulase activity in the mutant, indicating higher cellulase activity compared to the wild-type. Specifically, *SpGH9A3* exhibited stronger correlations with cellulose content and cellulase activity in the mutant leaves when compared to other genes in the family. Based on the data analysis above, it was found that *SpGH9A3* showed stronger correlation coefficients with cellulose content and cellulase activity in mutant leaves compared to other genes in the same family when wild was used as a control. As a result, further investigation including sequence analysis, gene cloning, and functional verification is planned for *SpGH9A3*, which has been provisionally identified as a potential candidate gene involved in the morphogenesis of mutants (Figure 6).

### 3.4. Bioinformatics Study of SpGH9A3 in Spathiphyllum

In the phylogenetic tree, the amino acid sequences of *AtKOR1* (*Arabidopsis*) (AAC83240.1), *AtGH9A1* (AED95850.1), *AtGH9A2* (NP_176738.1), and *AtGH9A4* (AEE77836.1) were found to be taxonomically congruent with *SpGH9A3* of *Spathiphyllum*. A multi-sequence alignment using DNAMAN 8 (version 7.0) software revealed that *SpGH9A3* shares a conserved *GH9* (glycoside hydrolase family 9) domain with other proteins (Figure S3).

The physicochemical characteristics of *SpGH9A3* were analyzed using ProtParam software (https://web.expasy.org/protparam/). The full-length cDNA of the *SpGH9A3* gene spans 858 bp and encodes 285 amino acids. The amino acid composition was predominantly alanine (211, 24.6%), glycine (230, 26.8%), and threonine (172, 20.0%). The theoretical isoelectric point (pI) of the protein was determined to be 8.79 with an approximate molecular weight of 31.7 KDa. A signal peptide sequence consisting of 29 amino acids and a cleavage site at VTT-EI was identified at the N-terminal of *SpGH9A3*. The secondary structure prediction of *SpGH9A3* protein revealed the presence of random coils (132, 46.32%), extended strands (58, 20.35%), α-helices (86, 30.18%), and β turns (9, 3.16%). The three-dimensional structure of *SpGH9A3* was analyzed using SWISS-MODEL, with a Global Model Quality Estimation (GMQE) score of 0.72, indicating a relatively stable protein structure. The predicted model showed a 77.15% similarity rate with the template, suggesting a reliable prediction. The gene structure of *SpGH9A3* mainly consists of α-helices and random coils, as supported by both secondary and tertiary structure predictions. These findings provide valuable insights into the function of *SpGH9A3*, paving the way for further research (Figure S4).

### 3.5. SpGH9A3 Protein Is Localized to the Cell Membrane

**Figure 7:**
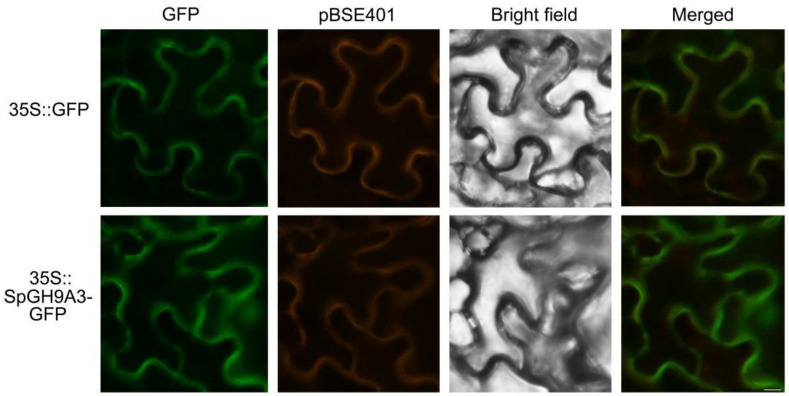
Subcellular localization of SpGH9A3. All images were captured with a fluorescence microscope. Scale bar = 20 μm.

The full-length SpGH9A3 protein was fused to GFP at the C-terminus and transiently expressed in *N. benthamiana* leaves under the control of the 35S promoter (35S::SpGH9A3-GFP) to investigate the localization of *SpGH9A3* in plant cells. The SpGH9A3-GFP fusion proteins were observed in the cell membrane of *N. benthamiana* leaves, indicating that *SpGH9A3* is localized to the cell membrane and suggesting that cellulose hydrolysis occurred in this site (Figure 7).

### 3.6. Overexpressing SpGH9A3 Changed the Leaf Morphology and Decreased Cellulose Content

An overexpression system utilizing the pCAMBIA1300 vector was employed to enhance the expression of *SpGH9A3* in *Arabidopsis*. The complete gene sequence of *SpGH9A3* was inserted into pCAMBIA1300 to generate the pCAMBIA1300-*SpGH9A3* vector, with wild-type *Arabidopsis* serving as the control group. Ten to fifteen *Arabidopsis* plants were inoculated with the vector.

**Figure 8:**
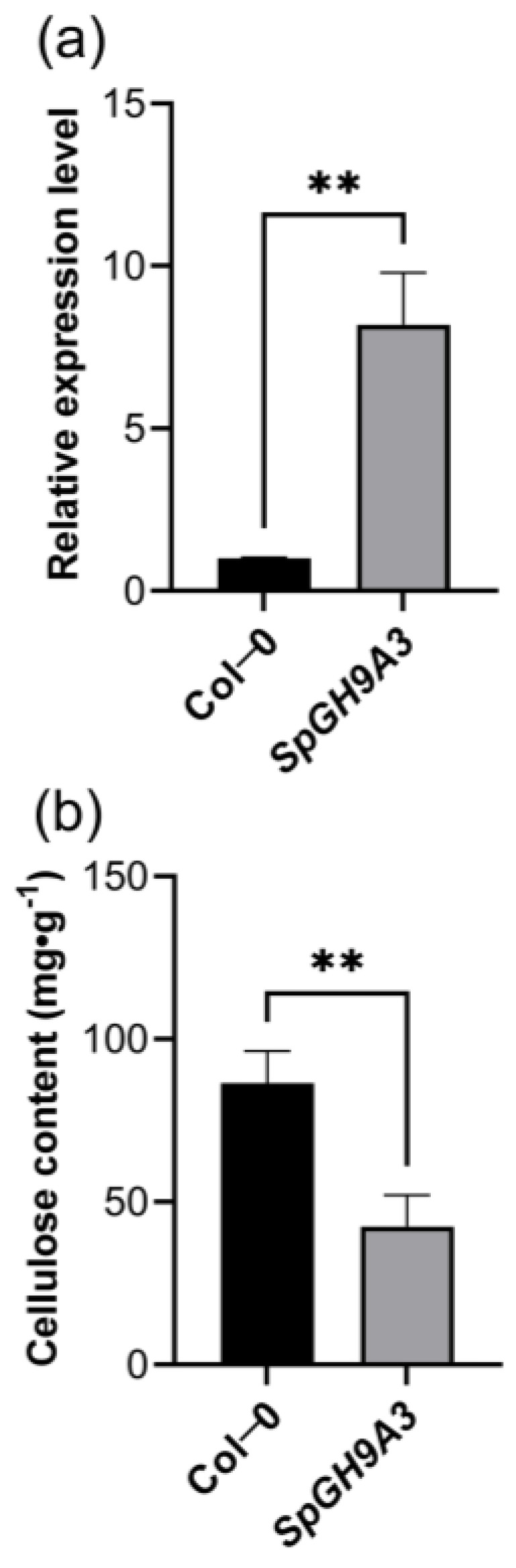
Relative expression levels and cellulose content of the overexpressed *SpGH9A3* gene in *Arabidopsis*. Using wild-type *Arabidopsis* leaves as a control, (**a**) represents the comparison of relative expression levels between wild-type and *SpGH9A3* overexpression plants, while (**b**) illustrates the comparison of cellulose content between wild-type and *SpGH9A3* overexpression plants. The asterisks indicate different levels of significant differences between clusters (**, *p* < 0.01).

After one month post-infection, transgenic plants displayed a notable increase in *SpGH9A3* expression, with leaves showing an 8-fold rise compared to the control group. Further examination of cellulose content in the leaves of transgenic *SpGH9A3* and wild-type plants revealed a significant decrease in cellulose content in the transgenic plants, accounting for only 49.00% of the control group (Figure 8).

**Figure 9:**
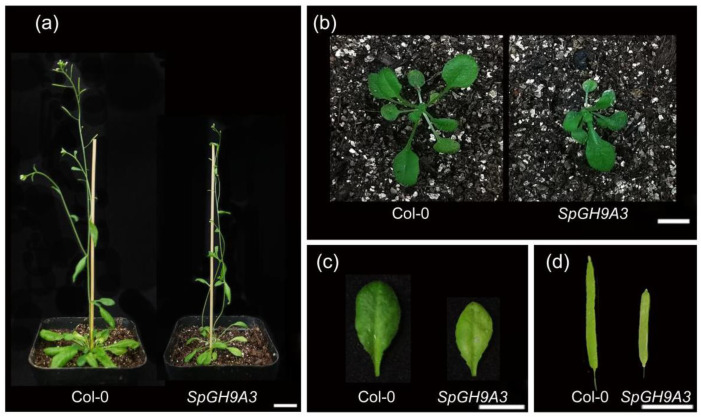
Phenotypic alterations caused by overexpression of *SpGH9A3* in *Arabidopsis*. (**a**–**d**) Comparative views of the Col-0 (left) and pCAMBIA1300-*SpGH9A3*-treated (right) plants, including lateral views of vegetative expansion stage (**a**), top-down views of vegetative growth stage (**b**), leaves (**c**), and siliques (**d**). Scale bars = 2 cm in (**a**), 1 cm in (**b**–**d**).

**Figure 10:**
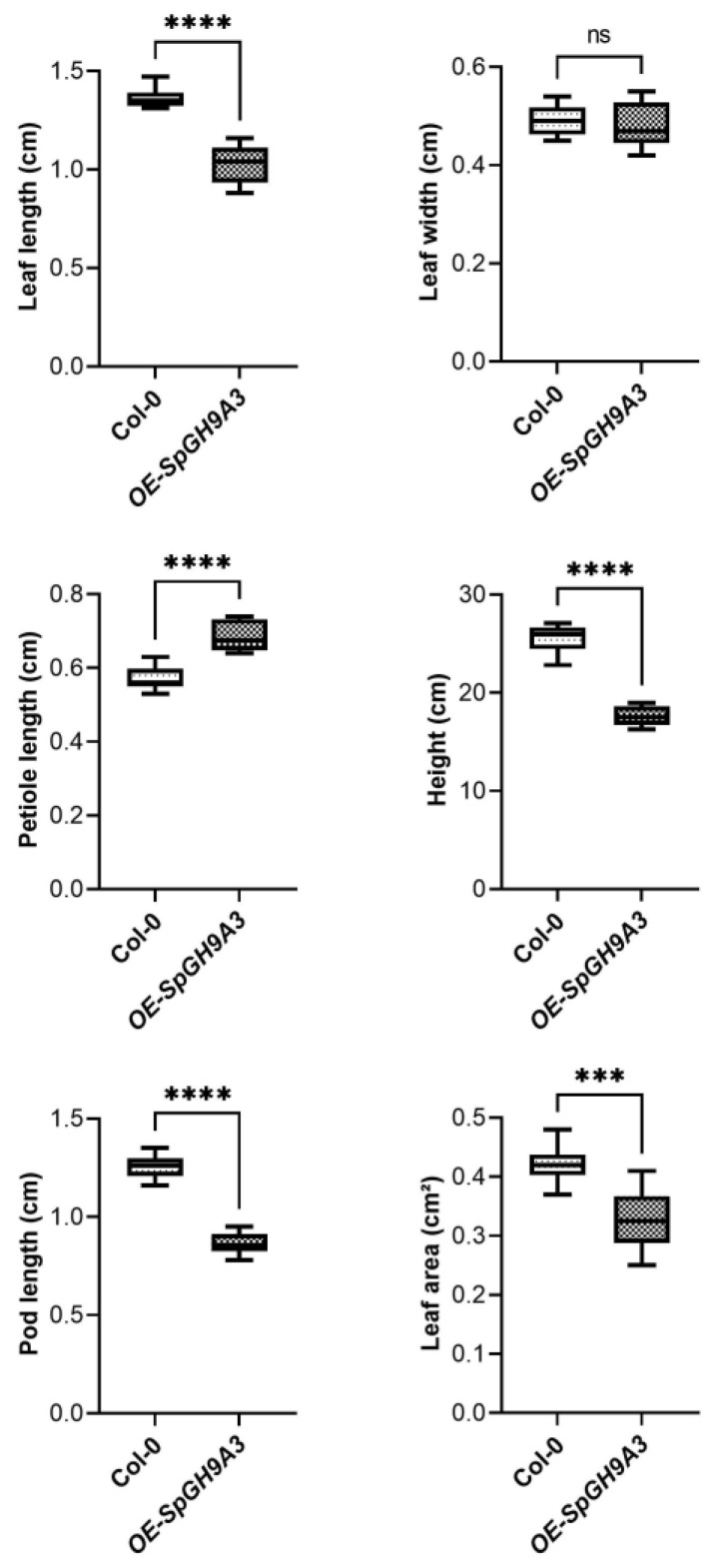
Leaf traits of overexpressed *SpGH9A3* gene in mature *Arabidopsis* leaves. Wild-type *Arabidopsis* leaves were used as controls. The asterisks indicate different levels of significant differences between clusters (ns, not statically significant; ***, *p* < 0.001; ****, *p* < 0.0001).

The statistical analysis revealed that overexpressing *SpGH9A3* plants exhibited significantly shorter leaf length, smaller leaf area, shorter pods, and shorter plants, amounting to 75%, 78%, 68%, and 69% of the control group, respectively. In contrast, leaf width remained unchanged at 98% of the control group with no observable differences. Additionally, there was a notable change in leaf morphology, with petiole length and leaf length–width ratio measuring 121% and 77% of the control group, respectively. These results suggest that the plant’s morphological characteristics, including leaves, pods, and overall plant structure, are greatly influenced by the overexpression of *SpGH9A3* (Figure 9 and Figure 10 and Table 2).

## 4. Discussion

Leaf morphology plays a crucial role in determining the aesthetic aspect of plants, with cell proliferation and cell expansion being key biological processes that influence it [25]. The growth and development of plants are closely tied to cellulose, a β-1,4-linked glucan that is a vital component of plant cell walls. Cellulose biosynthesis impacts cell expansion, division, mechanical strength, pathogen resistance, and various other aspects of plant growth [26]. The *GH9* family is essential in plant cellulose synthesis and hydrolysis [7]. The overexpression of *SpGH9A3* resulted in a decrease in leaf cellulose content, leading to a smaller leaf phenotype.

Three *GH9A* subfamily members, *SpGH9A1*, *SpGH9A2*, and *SpGH9A3* (GenBank: WYV98059.1), have been identified in *Spathiphyllum*. These genes display similar expression patterns during leaf growth and development, showing a high degree of sequence similarity and containing typical *GH9*-conserved domains. Previous studies have shown that cellulose is synthesized by the cellulose synthase complex, which is formed in the Golgi apparatus and then transported to the plasma membrane to build the cell wall structure [27,28]. The current study observed the presence of the SpGH9A3 protein on the cell wall, aligning with prior findings and suggesting that cellulose hydrolysis in *Spathiphyllum* leaves takes place within the cell wall.

The *GH9* family is a critical component of the hydrolase family involved in both cellulose synthesis and hydrolysis [29]. Mutations in the *KOR* gene have been observed to induce dwarfing in *Arabidopsis* plants and a significant reduction in cellulose content. Inhibition of *KOR* gene expression in *Arabidopsis* mutant plants also results in dwarf phenotypes and cell wall wrinkles, indicating the gene’s involvement in cellulose synthesis and modification [30]. Moreover, mutation of the *KOR* gene, which is homologous to *Cel3* in *Arabidopsis kor*-*1*, leads to organ shrinkage [31]. The overexpression of cottonwood *PdeKOR* in *Eucalyptus tenuifolia* promotes significant growth in stem segments [32]. Similarly, the expression of poplar endoglucanase in transgenic *Arabidopsis* plants accelerates growth and increases cellulose content [3]. Overexpressing the *Arabidopsis cel1* gene in poplar plants also enhances growth and cellulose content [33]. Mutations in *GH9A1* in *Arabidopsis* result in reduced cellulose content and play a crucial role in cellulose synthesis in the primary cell wall [13]. This study investigated the leaf phenotype following the overexpression and transformation of the *SpGH9A3* gene in *Arabidopsis* plants. The leaves of the overexpressed plants displayed a small-leaf phenotype, along with reduced growth and leaf cellulose content compared to the control. This experimental outcome slightly deviates from previous research, possibly due to the stringent regulation of cellulase activity across different species and stages of transcription, translation, and post-translation [34].

Morphological differences were observed in the leaves of wild and mutant plants, with varying cellulose content and cellulase activity trends. Transcriptome analysis revealed significant differences in the expression levels of cellulase *SpGH9s*. While the cellulase activity of mutant leaves was similar to wild leaves during certain stages, it notably increased in the early spread stage.

Further verification of the potential functions of *SpGH9s* in *Spathiphyllum* using additional molecular biology techniques and tools is essential. These findings lay the groundwork for further exploration of *SpGH9s*, providing fresh insights into its biological functions. The intricate chemical basis and regulatory mechanisms of cell wall polysaccharide biosynthesis remain complex and warrant further comprehensive investigation. To enhance our comprehension, a combination of experimental and computational approaches should be employed to isolate target proteins, develop enzyme analysis methods, and construct multi-omics regulatory networks to establish detailed molecular models.

## 5. Conclusions

This study focused on *Spathiphyllum* ‘Mojo’ and its mutant leaves as research materials, with morphological index measurements revealing significant differences between the two types of leaves. Four *GH9* family genes were identified from the transcriptome data of *Spathiphyllum*. By analyzing the correlation coefficient between gene expression, leaf cellulose content, and cellulase activity, the study preliminarily identified *SpGH9A3* as the most relevant gene for the expasion of mutant leaves. Overexpression of the *SpGH9A3* gene in transformed *Arabidopsis* plants resulted in smaller leaves and reduced leaf cellulose content compared to the control group. These findings suggest that differential expression of the *SpGH9A3* gene in the leaves of different *Spathiphyllum* varieties may impact cellulase activity, subsequently influencing leaf cellulose content at various stages of expansion and ultimately leading to differences in leaf morphology. This research enhances our comprehension of the roles of glycoside hydrolase family genes and lays a foundation for future investigations.

## Figures and Tables

**Table 1:** The primary characteristics of *Spathiphyllum* leaf growth at three stages.

Stage	Number	Primary Growth and Morphological Characteristics
The curled leaf stage	W1/M1	Approximately ten days after the leaf buds transition to white, the tips of the leaves gradually change from white to yellow/white, while the base of the leaves remains white. During this period, curled leaves develop, with the leaves being enveloped in leaf sheaths.
The early spread stage	W2/M2	Approximately 10 to 13 days after the formation of curled leaves, the leaves emerge from the leaf sheath and begin to gradually unfold. During this process, the leaf color transitions from yellow/white to a delicate green.
The late spread stage	W3/M3	Approximately ten days after the onset of leaf color change, the leaves transition from light green to a deeper green. During this period, the leaves gradually expand, completing their growth. At this stage, the characteristics of the leaves stabilize and exhibit no further changes.

W—wild; M—mutant.

**Table 2:** Growth variation in leaves with overexpression of the *SpGH9A3* gene.

	WT	OE of *SpGH9A3*	*SpGH9A3*/WT (%)
Leaf length (cm)	1.36 ± 0.05	1.02 ± 0.10 *	75.00%
Leaf width (cm)	0.49 ± 0.03	0.48 ± 0.05	97.96%
Length–width ratio	2.78 ± 0.19	2.14 ± 0.28 *	76.98%
Petiole length (cm)	0.57 ± 0.03	0.69 ± 0.04 *	121.05%
Leaf area (cm^2^)	0.42 ± 0.03	0.33 ± 0.05 *	78.57%
Silique length (cm)	1.25 ± 0.06	0.86 ± 0.06 *	68.80%
Plant height (cm)	25.49 ± 1.42	17.63 ± 1.00 *	69.16%

The asterisks indicate different levels of significant differences between clusters (ns, not statically significant; *, *p* < 0.05).

## Data Availability

All materials and related data in this study are provided in the Supplementary Materials.

## Supplementary Materials

The following supporting information can be downloaded at: https://www.mdpi.com/article/10.3390/genes15091132/s1, Figure S1: Comparison of leaf morphology differences between *Spathiphyllum* ‘Mojo’ and ‘Mojo’-Ssm-1. Figure S2: KEGG annotation results of three expasion stages of *Spathiphyllum* leaves. Figure S3: Multiple amino acid sequence alignment analysis of *SpGH9A3* and *Arabidopsis GH9A* subfamily genes. Figure S4: Bioinformatics analysis of *SpGH9A3*. Table S1: Comparison of leaf morphology indexes. Table S2: Reagents used in experiments. Table S3: Equipment used in experiments.
