# Supplementary material for: Genetic Evidence of SpGH9A3 in Leaf Morphology Variation of Spathiphyllum ‘Mojo’
Source: Genes (Basel). 2024 Aug 28;15(9):1132. doi: 10.3390/genes15091132 (PMC11431335; doi:10.3390/genes15091132)
Supplement: Supplementary file 1 [file genes-15-01132-s001.zip › Supplementary Table.pdf]

Table S1 Comparison of leaf morphology indexes

	Spathiphyllum 'Mojo'	Spathiphyllum 'Mojo'-Ssm-1	Mojo/Ssm-1 （%）
Leaf length (cm)	15.89±0.66	11.00±0.69*	144.45%
Leaf width (cm)	4.08±0.13	5.74±0.26*	71.08%
Length-width ratio	3.90±0.21	1.92±0.21*	203.13%
Petiole length (cm)	14.97±0.62	8.75±0.28*	171.09%
Leaf area (cm2)	41.19±2.10	38.19±1.08*	107.86%
Plant height (cm)	26.68±0.66	22.98±0.85*	116.10%
Crown width (cm)	41.47±1.39	38.18±1.08*	108.62%
Leaf shape	Lanceolate	Ovate	
Leaf color	Light green	Dark green	

Table S2 Reagents used in experiments

Name	Model specifications	The Place Of Origin
Hipure Plant RNA Mini kit	R4151	Guangzhou Magen Biotechnology Co., Ltd.
HiScript IV RT SuperMix for PCR	R312-01	Nanjing Vazyme Biotechnology Co., Ltd.
HiScript IV RT SuperMix for qPCR	R423-01	Nanjing Vazyme Biotechnology Co., Ltd.
Plasmid extraction kit	P1001C	Guangzhou Magen Biotechnology Co., Ltd.
2×SuperTaq PCR StarMix	A003	Beijing GenStar Biotechnology Co., Ltd.
TSINGKE TSV-S3 Trelief Seamless Cloning Kit	TSV-S3	Beijing Tsingke Biotech Co., Ltd.
DNA Marker	M022	Beijing Tsingke Biotech Co., Ltd.
DH5α Competent E.coli Strain	C502	Nanjing Vazyme Biotechnology Co., Ltd.
GV3101 Agrobacterium Strain	LM12-162	Shanghai LMAI Biotech Engineering Co., Ltd.
Taq pro universal SYBR qPCR master mix	Q712-02	Nanjing Vazyme Biotechnology Co., Ltd.
Cellulose (CLL) content detection kit	BC4280	Beijing Solarbio Science & Technology Co., Ltd.
Cellulase (CL) activity detection kit	BC2545	Beijing Solarbio Science & Technology Co., Ltd.

Table S3 Equipment used in experiments

Name	Model specifications	The Place Of Origin
Desktop high speed centrifuge.	5810D	Eppendorf Corporate, German
Desktop high speed refrigerated centrifuge	5810R	Eppendorf Corporate, German
Spectrophotometer.	RS232C	Eppendorf Corporate, German
Nucleic acid electrophoresis instrument	EQ2701	LABNET, USA
MyCycler Thermal Cycler System	T100	Bio-Rad Laboratories, Inc., USA
Real-time fluorescence quantitative Thermal Cycler System.	Bio-Rad Connect	Bio-Rad Laboratories, Inc., USA
Gel imaging system.	T2A	Bio-Rad Laboratories, Inc., USA
Electronic analytical balance.	BSA224S	Sartorius, German
High pressure automatic sterilization pot	SS325	TOMY, Japan
Ultra low temperature refrigerator	702REL1	Thermo Scientific, USA
Full wavelength microplate reader	A51119500C	Thermo Scientific, USA
Confocal laser microscopy.	LSM800	Zeiss, German
