# Supplementary figures and images for: Genetic Evidence of SpGH9A3 in Leaf Morphology Variation of Spathiphyllum ‘Mojo’
Source: Genes (Basel). 2024 Aug 28;15(9):1132. doi: 10.3390/genes15091132 (PMC11431335; doi:10.3390/genes15091132)

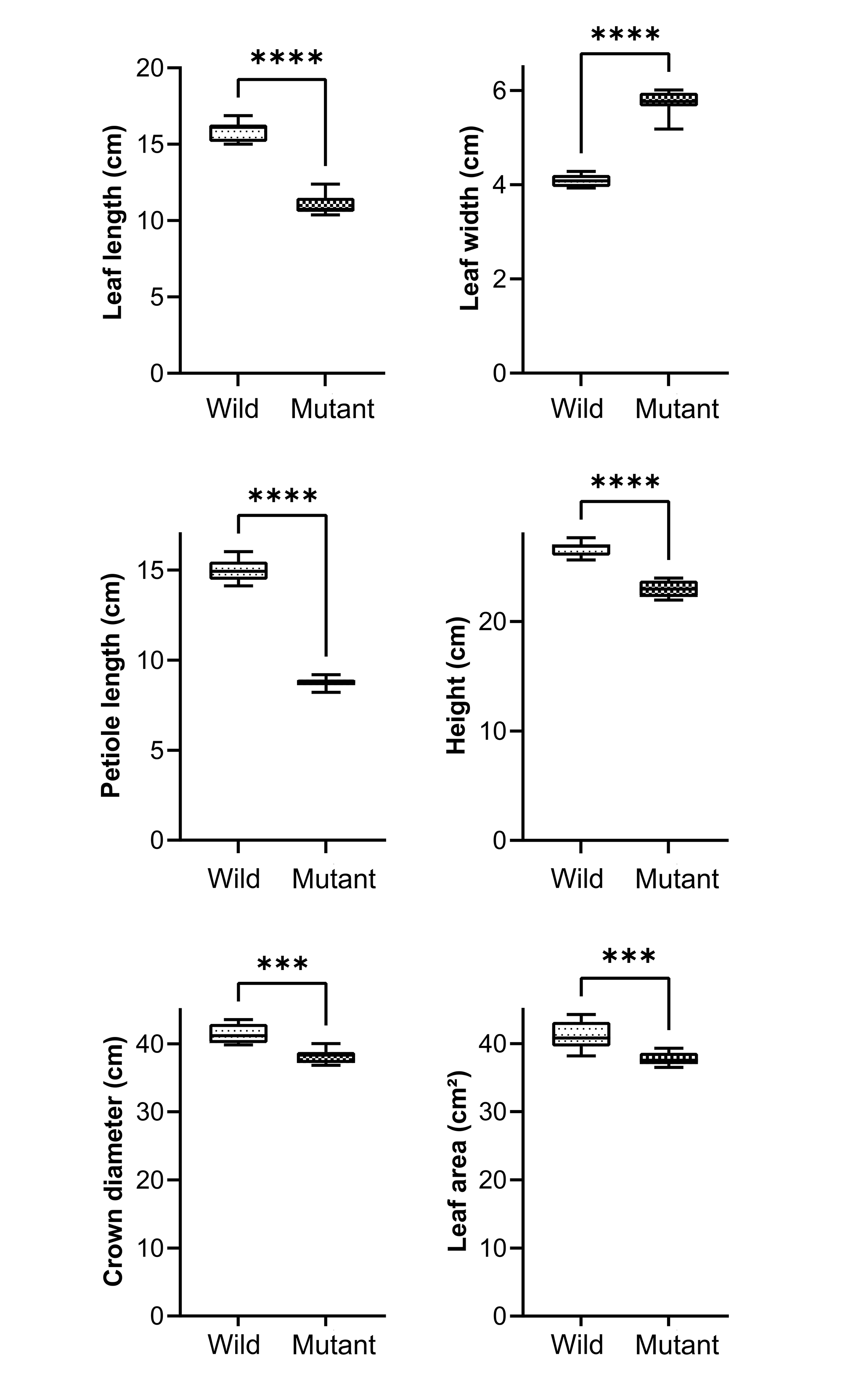

Supplement: Supplementary file 1 [file genes-15-01132-s001.zip › S1.png]

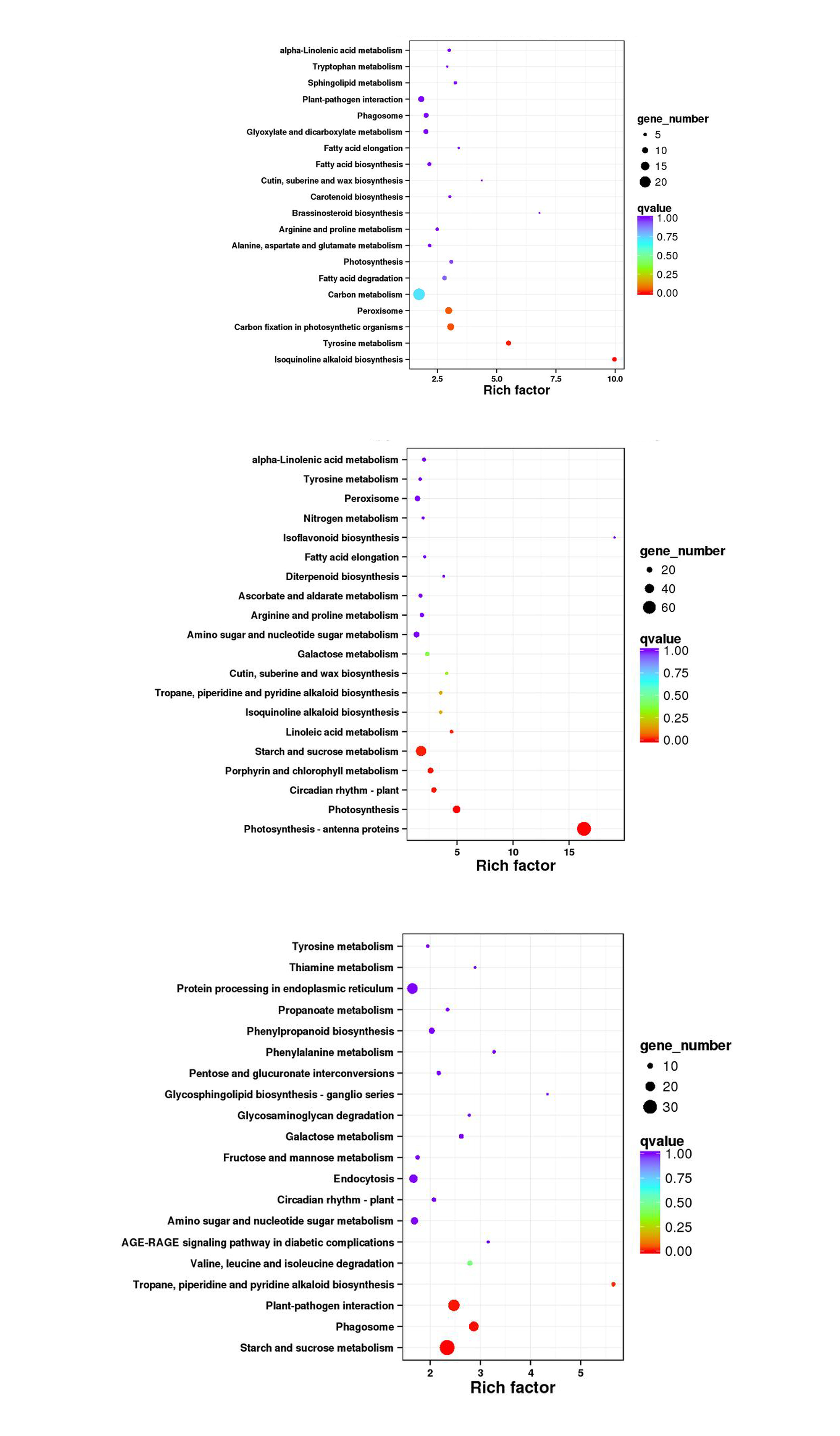

Supplement: Supplementary file 1 [file genes-15-01132-s001.zip › S2.png]

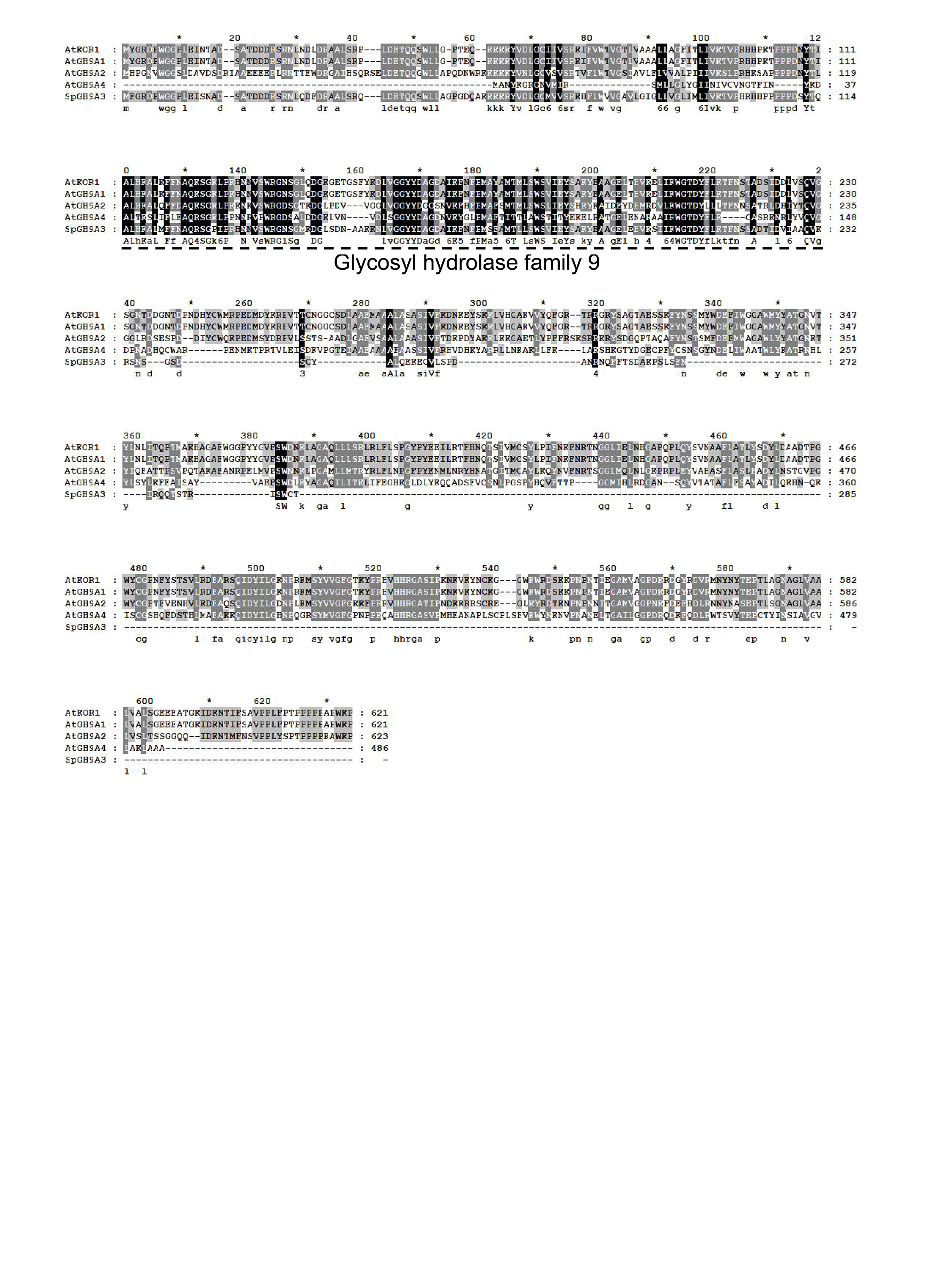

Supplement: Supplementary file 1 [file genes-15-01132-s001.zip › S3.png]

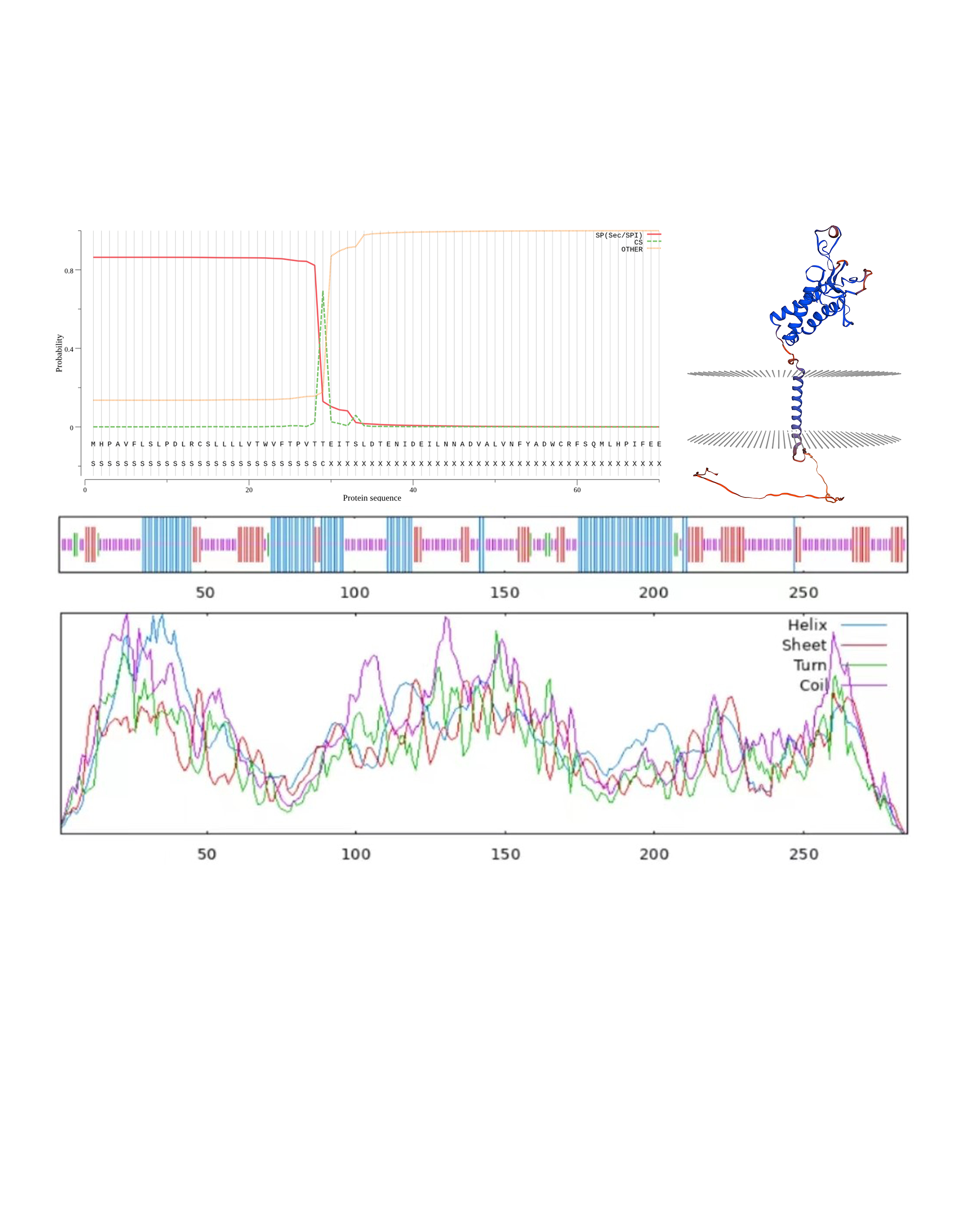

Supplement: Supplementary file 1 [file genes-15-01132-s001.zip › S4.png]
